# 3D electron diffraction analysis of a novel, mechanochemically synthesized supramolecular organic framework based on tetra­kis-4-(4-pyridyl)phenyl­methane

**DOI:** 10.1107/S2052520623007680

**Published:** 2023-09-26

**Authors:** Danilo Marchetti, Alessandro Pedrini, Chiara Massera, Moussa Diame Faye Diouf, Christian Jandl, Gunther Steinfeld, Mauro Gemmi

**Affiliations:** a Dipartimento di Scienze Chimiche, della Vita e della Sostenibilità Ambientale, University of Parma, Parco Area delle Scienze 17/A, Parma, 43123, Italy; b Center for Materials Interfaces, Electron Crystallography, Istituto Italiano di Tecnologia, Viale Rinaldo Piaggio 34, Pontedera, 56025, Italy; c ELDICO Scientific AG, PARK INNOVAARE: deliveryLAB, Villigen, 5234, Switzerland; University of Antwerp, Belgium

**Keywords:** 3D electron diffraction, electron diffractometer, mechanochemistry, supramolecular organic frameworks

## Abstract

Tetra­kis-4-(4-pyridyl)­phenyl­methane (TPPM) is a tetrahedral rigid molecule that crystallizes forming a dynamically responsive supramolecular organic framework (SOF). When exposed to different stimuli, this supramolecular network can reversibly switch from an empty to a filled solvated solid phase. This article describes a novel expanded form of a TPPM-based SOF that has been mechanochemically synthesized and whose crystal structure has been determined by 3D electron diffraction analysis using a novel electron diffractometer.

## Abstract 2

A new supramolecular organic framework (SOF) based on the tetrahedral rigid molecule tetra­kis-4-(4-pyridyl)­phenyl­methane has been mechanochemically synthesized. The crystal structure of the SOF containing benzyl alcohol has been determined using 3D electron diffraction analysis with a novel electron diffractometer.

## 1. Introduction

**Figure 1:**
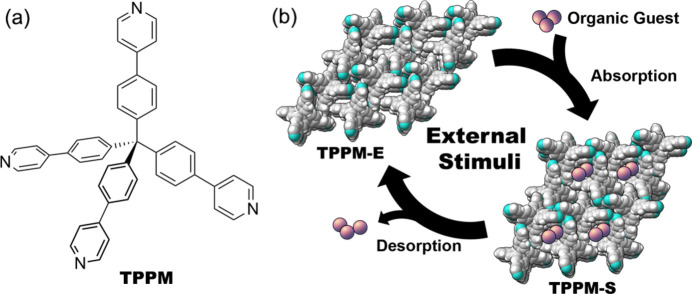
(*a*) Molecular sketch of tetra­kis-4-(4-pyridyl)­phenyl­methane (TPPM). (*b*) Schematic representation of the stimuli-responsive behaviour of the TPPM-based framework.

Supramolecular organic frameworks (SOFs) are an important class of functional porous materials which have been extensively studied in recent years due to their possible applications in sensing and molecular recognition (Ishi-i *et al.*, 2020 ▸; Chen *et al.*, 2022 ▸), as well as for their dynamic behaviour upon application of external stimuli (Natarajan *et al.*, 2013 ▸; Wang *et al.*, 2019 ▸). We have recently reported the selective and reversible solvent uptake by an SOF based on tetrakis-4-(4-pyridyl)­phenyl­methane (TPPM), a rigid, aromatic tecton [Fig. 1 ▸(*a*)] which undergoes a single-crystal-to-single-crystal transformation when exposed to vapours of selected organic solvents and heat (Marchetti *et al.*, 2022 ▸). As represented in Fig. 1 ▸(*b*), this SOF exists in two forms: an empty, closed structure containing only small, isolated voids (TPPM-E), and an expanded framework endowed with channels that could be occupied by different guests (chloro­form, ethanol, benzene, aceto­nitrile *etc*.). In order to obtain the solvated, expanded form (TPPM-S), the empty form can be exposed to vapours of specific organic solvents (*e.g.* chloro­form and benzene) for a few minutes. Another way to obtain the expanded form consists of crystallizing the tecton by slow evaporation of a solution with the desired solvent, typically chloro­form. Chloro­form can then be displaced by soaking the crystals for a couple of days in a different medium, preferably one in which TPPM shows very low solubility (such as, for example, ethanol or aceto­nitrile).

An alternative approach is the use of mechanochemistry, an environmentally friendly synthetic strategy that has been shown to be green, versatile and efficient (Howard *et al.*, 2018 ▸; Gomollón-Bel, 2019 ▸; Ardila-Fierro & Hernández, 2021 ▸). Mechanochemistry often yields microcrystalline materials that cannot be analysed with single-crystal X-ray diffraction methods. While in the past this was a severe obstacle, nowadays it has been demonstrated that this problem can be overcome by using 3D electron diffraction (ED) directly on the crude reaction product (Marchetti *et al.*, 2021 ▸; Biswas *et al.*, 2023 ▸; Gogoi *et al.*, 2023 ▸; Sasaki *et al.*, 2023 ▸).

The development of 3D ED has been impressive in the last decade, becoming a method well established for the structure determination of nanocrystalline compounds (Gemmi *et al.*, 2019 ▸). Initially, 3D ED was mainly applied to inorganic beam-resistant materials (Mugnaioli *et al.*, 2009 ▸), but, with the introduction of continuous-rotation methods (Nannenga *et al.*, 2014 ▸; Gemmi *et al.*, 2015 ▸) combined with fast, single-electron detectors (Nederlof *et al.*, 2013 ▸), it has also become a valuable technique for radiation-sensitive materials like hybrid (Huang *et al.*, 2021 ▸), organic (Andrusenko & Gemmi, 2022 ▸; Andrusenko *et al.*, 2023 ▸) and macromolecular compounds (Xu *et al.*, 2019 ▸). This is having an indirect influence also on synthetic chemistry, since by widening the spectrum of crystalline materials whose crystal structure can be investigated, we are able to explore synthesis routes which up to now have remained extremely challenging, such as mechanochemistry.

The increased interest caused by the successes of 3D ED has also triggered the development of a new class of instruments dedicated to 3D ED as an alternative to the existing solutions based on transmission electron microscope architectures. The ELDICO ED-1 is a new electron diffractometer specifically designed to collect 3D ED data in a reliable and reproducible way (Simoncic *et al.*, 2023 ▸). In this paper, we present the structure solution and refinement of a SOF having channels partially filled with solvent, one of the first unknown structures obtained from this instrument. Other very recent examples have been reported by Sieger *et al.* (2023 ▸) and Woods *et al.* (2023 ▸).

## 2. Methods

### 2.1. Mechanochemical synthesis

In order to obtain the solvated form of the TPPM-based SOF, crystals of the empty form (TPPM-E; obtained by heating a batch of single crystals of TPPM·CHCl_3_ at 100°C for 2 h) were manually ground in the presence of benzyl alcohol (BnOH). The reaction consists of a liquid-assisted grinding (LAG) in which one of the reagents (BnOH) also acts as a liquid additive.

### 2.2. 3D ED data collection

The ELDICO ED-1 electron diffractometer (see Fig. S2 in the supporting information) is a novel instrument designed specifically for easy access to diffraction experiments and not primarily for imaging (like a transmission electron microscope). This results in a horizontal setup in which the sample rotates around a vertical axis and the number of lenses is limited to just the illumination and scanning system. The diffractometer is assembled from three crucial components: the electron-beam system, the goniometer and a single-electron detector. A detailed description of each can be found in the supporting information.

The nanocrystalline powder was directly characterized, as-synthesized, through 3D ED analysis. The sample was prepared with the procedure reported in the supporting information. The data collection was conducted on the ELDICO ED-1 in continuous-rotation mode with a beam diameter of ∼750 nm. This modality is analogous to a single-crystal X-ray experiment with an area detector; however, in the case of electrons, the much stronger interaction with matter allows a faster data collection on remarkably smaller crystal volumes compared with X-rays. The 3D ED experiments lasted only a few minutes. On the other hand, the use of very sensitive and fast single-electron detectors like the Dectris QUADRO does not require an intense beam; therefore the total electron dose is minimized (Gemmi & Lanza, 2019 ▸).

The high mechanical stability of the vertical goniometer guarantees that, after a fine optimization of the eucentric height, the nanocrystal is maintained in a stable position inside the electron beam during the whole rotation.

Scanning transmission electron microscopy (STEM) imaging was used to search for crystals suitable for the 3D ED analysis and their crystal quality was preliminarily checked with a single diffraction pattern, placing the beam on the crystal of interest.

**Figure 2:**
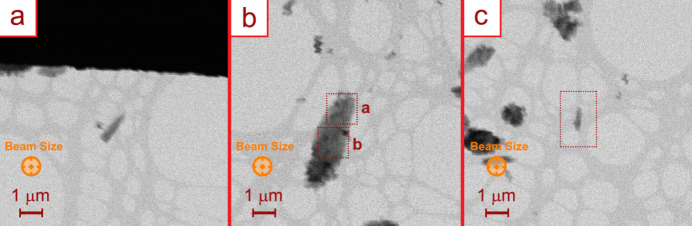
STEM images of the three TPPM·BnOH microcrystals used for the 3D ED data collection. (*a*) Crystal 1, (*b*) crystal 2. The dotted squares highlight the two areas in which data collection 2^*a*^ and 2^*b*^ were conducted. (*c*) Crystal 3.

Three different microcrystals were characterized in order to maximize the completeness (Fig. 2 ▸). Despite the TPPM-based crystals being quite sensitive to the electron beam, as previously checked on a standard transmission electron microscope, the electron diffractometer configuration allowed a complete 3D ED experiment. Indeed, ED frames were collected over 109° of reciprocal space, with a resolution of 1.2 Å, avoiding the amorphization of the crystals or any visible resolution loss at the end of the experiment (Fig. S3). For the three analysed crystals, four data sets were collected. The *PETS2* software (Palatinus *et al.*, 2019 ▸) was employed to analyse the diffraction patterns to find the unit cell, index and integrate the reflection intensities.

The collected diffraction data for each crystal were combined in one single data set using the merging tool implemented in the *PETS2* software. The merged data set was employed for the *ab initio* structure determination, performed by standard direct methods using the *SHELXT* software (Sheldrick, 2015 ▸). The data were initially refined with a fully kinematical approximation and the least-squares refinement was performed with the software *SHELXL-2014* interfaced with *Olex2* (Dolomanov *et al.*, 2009 ▸).

For the dynamical refinement, the diffraction data coming from different crystals were analysed separately with *PETS2*. The reflections were properly integrated considering a rotation semiangle (Δα) of 0.25° (corresponding to half of the angular integration step) and the integrated intensities were combined in virtual frames (see the supporting information). The dynamical refinement was carried out using the *Jana2020* software (Petříček *et al.*, 2014 ▸) simultaneously on different data sets (see Table S3).

### 2.3. Powder X-ray diffraction

The powder X-ray diffraction (PXRD) patterns of the samples were collected using Ni-filtered Cu *K* radiation [λ(*K*α1) = 1.5406 Å, λ(*K*α2) = 1.5444 Å] on a Rigaku SmartLab XE diffractometer equipped with a HyPix-3000 detector. The data were processed with *SmartLab Studio II* (Rigaku). PXRD patterns were collected in Bragg–Brentano geometry in the 2θ range 5–75°, with the sample placed on a silicon zero-background specimen holder. The LeBail refinement on PXRD data was conducted with *Jana2020* (see the supporting information).

## 3. Results

**Figure 3:**
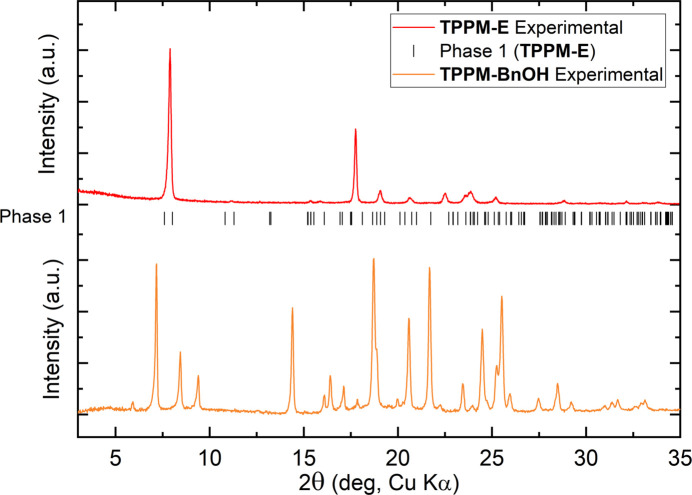
Powder X-ray profile of the TPPM empty form (red line) and calculated reflections (black sticks), compared with the mechanochemical product (orange line).

The mechanochemical product is crystalline; however, its PXRD profile cannot be indexed as the SOF empty form (Fig. 3 ▸).

A probable strong preferred orientation and the low resolution of X-ray diffraction data hamper a structure solution based on PXRD characterization and prompted us to try 3D ED. The compound was stable under the ELDICO ED-1 beam and full data sets could be automatically collected on it.

**Figure 4:**
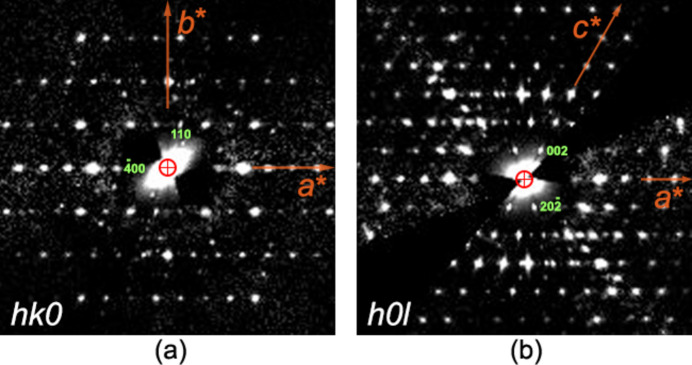
Section of reciprocal space reconstructed with *PETS2* from the 3D ED data. (*a*) *hk*0 reciprocal plane section showing the extinction rule *h* + *k* = 2*n*. (*b*) *h*0*l* reciprocal plane section showing the angle β* ≃ 61° and the reflection condition for the *c*-glide plane, *l* = 2*n*. The reciprocal-space sections are calculated on the merged data sets of crystals 1, 2 and 3.

Despite the presence of some spurious reflections coming from other small crystals falling inside the illuminated area, the reconstructed reciprocal space (Fig. 4 ▸) could be unambiguously indexed with a monoclinic *C*-centred lattice with parameters *a* = 27.946 (7), *b* = 7.0236 (5), *c* = 21.610 (3) Å, β = 119.031 (14)°. The *C*-centring extinction rule and the *c* glide clearly observed in the *h*0*l* plane led to the identification of the *C*1*c*1 extinction symbol compatible with space groups *Cc* and *C*2/*c*. The indexed data were then integrated with the fit profile model of *PETS2*, in which the mosaicity and resolution dependence of the peak width were refined globally, and then the orientation of each pattern (geometrical optimization) was optimized (Fig. S4).

The correct unit-cell determination was checked on PXRD data through a LeBail refinement (Fig. S1). The refinement converges to slightly bigger unit-cell parameters with respect to those obtained from the ED data (Table S4). This result can be ascribed to a loss of solvent molecules due to the strong vacuum condition inside the electron diffractometer column. It is well known that porous materials, like MOFs (metal–organic frameworks) and SOFs, have the capability to release the guest molecules held inside their pores, and this process is accelerated by vacuum and heat. Indeed, under the previously mentioned stimuli, the expanded, solvated form of the TPPM-based SOF could easily release guest molecules, such as CHCl_3_, EtOH, C_6_H_6_ and CH_3_CN (Marchetti *et al.*, 2022 ▸). However, the fact that the unit-cell volume remains significantly larger than that of the empty phase (Table S4) indicates that the BnOH release is only partial and the molecules remaining in the pores can stabilize the expanded form of the TPPM-based SOF also under high vacuum [∼10^−7^ mbar [1 mbar = 0.1 kPa)] conditions. This is quite unusual for this SOF since with all other solvents the only phase detected in high vacuum was the empty one.

**Figure 5:**
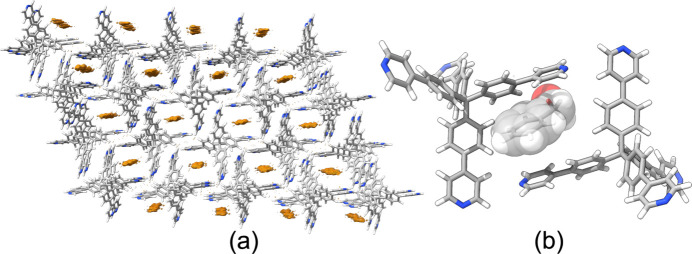
(*a*) Difference Fourier map calculated on the TPPM supramolecular network free of guest molecules. The electrostatic potential is reported as an orange surface {isosurface level 2.5σ[Δ*V*(*r*)]}, with the nitro­gen atoms in blue, carbon atoms in light grey and hydrogen atoms in white. (*b*) View of the benzyl alcohol guest packed between two TPPM molecules in the TPPM·0.5BnOH crystal phase. For clarity, only one of the two possible orientations of the BnOH molecule is displayed. The oxygen atoms are represented in red, carbon atoms in grey, nitro­gen atoms in blue and hydrogen atoms in white. The solvent molecule is shown in space-filling mode.

The data sets collected from the analysed crystals were merged (using the merging procedure of *PETS2*), and the structural model was then solved *ab initio* with standard direct methods in space group *C*2/*c*. The model was initially refined kinematically, *i.e.* with the measured intensity considered proportional to the square modulus of the structure factor. The kinematically refined model revealed the formation of a supramolecular network characteristic of the SOF expanded form (Marchetti *et al.*, 2022 ▸), in which the TPPM molecules are mainly involved in CH⋯N interactions (Figs. S8 and S9). The existence of BnOH molecules inside the pores was highlighted by the difference Fourier map calculation, which shows the presence of residual electrostatic potential in the framework channels [Fig. 5 ▸(*a*)]. The modelling of the guest molecules embedded in the SOF channels required them to be disordered over two equivalent positions, each with occupancy of approximately 0.25 (Fig. S6); they were then modelled as rigid-body molecules, in a ratio of 1:2 with respect to TPMM. The solvent stoichiometry calculated from thermogravimetric and NMR analysis over the crude product show a ratio of TPPM and BnOH near to 1:1 (Figs. S11 and S13). This result confirms that, during the diffraction experiment, a partial removal of BnOH has taken place under vacuum, as suspected from the moderate decrease in the lattice parameters detected with respect to PXRD data.

**Figure 6:**
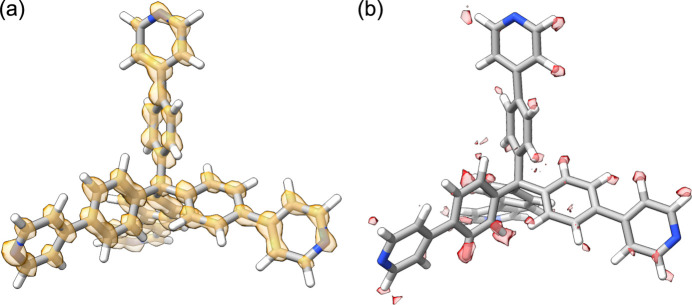
(*a*) Superposition between the calculated potential map and the TPPM molecule in the dynamically refined phase of TPPM·0.5BnOH. (*b*) Superposition between the difference potential map, calculated from the structural model without H atoms, and the TPPM molecule from the TPPM·BnOH structure after the dynamical refinement. The Fourier map and difference map are represented with an isosurface level of 2σ[Δ*V*(*r*)].

To improve the TPPM·0.5BnOH structure and have better agreement factors between the experimental and calculated intensities, a dynamical refinement, which takes into account multiple scattering effects through a full Bloch calculation (Palatinus *et al.*, 2015 ▸) is needed. However, if we want to keep the high coverage obtained by merging data from different crystals, we are obliged to perform the refinement against multiple data sets, one for each crystal, since for each different crystal the dynamical effects will be different due to their different thickness. We considered three different crystal data sets (crystals 2^*a*^, 2^*b*^, 3) and refined the structure dynamically against these three data sets simultaneously. Remarkably, the ED data quality proved suitable for a dynamical refinement and the refinement successfully converged. The dynamical refinement requires one to consider and refine, together with the structural model, the thickness of the sample at 0° of tilt. In the case of multiple data sets, the number of refined thicknesses equals the number of crystals involved (in our case crystal 2^*a*^ 645 Å, crystal 2^*b*^ 578 Å, crystal 3 738 Å). The dynamical data treatment allowed, as expected, a concrete reduction of the agreement factors: the *R*_1_(obs) value was reduced from 27.47% to 14.42%. The atomic displacement parameters were also refined without any restraints, leading to a structural model that better represents the mobility of the pyridyl rings with respect to the inner phenyl groups (Fig. S10). From the calculation of the difference Fourier map, it was also possible to detect the positions of 80% of the hydrogen atoms of the TPPM molecule (Fig. 6 ▸).

## 4. Conclusions

A new expanded phase of a TPPM-based SOF was synthesized by mechanochemical synthesis. The crystal structure of the as-synthesized product was elucidated by 3D ED analysis with a novel electron diffractometer. This is one of the first structures solved with a horizontal electron diffractometer with a completely new design with respect to a standard transmission electron microscope. The data quality allowed us to solve the crystal structure of the SOF, leading to the discovery that the vacuum inside the instrument was not enough to completely empty the channels. The residual BnOH molecules in the channels could be detected in the difference Fourier maps and their position properly refined. The structure could also be refined dynamically against multiple data sets collected on three crystals. To our knowledge, this is one of the first successful attempts at such a procedure.

## 5. Related literature

The following references are cited in the supporting information: Friščić *et al.* (2009 ▸), Garbuglia & Steinfeld (2022 ▸), Kitagawa *et al.* (2013 ▸), Niebel *et al.* (2021 ▸), Sheldrick (2008 ▸).

## Supplementary Material

Crystal structure: contains datablock(s) I. DOI: 10.1107/S2052520623007680/je5052sup1.cif

Structure factors: contains datablock(s) I. DOI: 10.1107/S2052520623007680/je5052Isup2.hkl

Supporting information including Tables S1-S6 and Figs. S1-S14. DOI: 10.1107/S2052520623007680/je5052sup3.pdf

CCDC reference: 2292774

## References

[bb1] Andrusenko, I. & Gemmi, M. (2022). *WIREs Nanomed Nanobiotechnol.* **14**, e1810. 10.1002/wnan.1810 PMC9539612 35595285

[bb2] Andrusenko, I., Hall, C. L., Mugnaioli, E., Potticary, J., Hall, S. R., Schmidt, W., Gao, S., Zhao, K., Marom, N. & Gemmi, M. (2023). *IUCrJ*, **10**, 131–142. 10.1107/S205225252201154X PMC9812223 36598508

[bb3] Ardila-Fierro, K. J. & Hernández, J. G. (2021). *ChemSusChem*, **14**, 2145–2162. 10.1002/cssc.202100478 33835716

[bb4] Biswas, S., Banerjee, S., Shlain, M. A., Bardin, A. A., Ulijn, R. V., Nannenga, B. L., Rappe, A. M. & Braunschweig, A. B. (2023). *Faraday Discuss.* **241**, 266–277. 10.1039/d2fd00122e PMC10088556 36134559

[bb5] Chen, C., Guan, H., Li, H., Zhou, Y., Huang, Y., Wei, W., Hong, M. & Wu, M. (2022). *Angew. Chem. Int. Ed.* **61**, e202201646. 10.1002/anie.202201646 35352465

[bb6] Dolomanov, O. V., Bourhis, L. J., Gildea, R. J., Howard, J. A. K. & Puschmann, H. (2009). *J. Appl. Cryst.* **42**, 229–341.

[bb50] Friščić, T., Childs, S. L., Rizvi, S. A. A. & Jones, W. (2009). *CrystEngComm*, **11**, 418–426.

[bb7] Garbuglia, F. & Steinfeld, G. (2022). European Patent Office. EP22192609.0.

[bb8] Gemmi, M. & Lanza, A. E. (2019). *Acta Cryst.* B**75**, 495–504. 10.1107/S2052520619007510 32830707

[bb9] Gemmi, M., La Placa, M. G. I., Galanis, A. S., Rauch, E. F. & Nicolopoulos, S. (2015). *J. Appl. Cryst.* **48**, 718–727.

[bb10] Gemmi, M., Mugnaioli, E., Gorelik, T. E., Kolb, U., Palatinus, L., Boullay, P., Hovmöller, S. & Abrahams, J. P. (2019). *ACS Cent. Sci.* **5**, 1315–1329. 10.1021/acscentsci.9b00394 PMC6716134 31482114

[bb11] Gogoi, D., Sasaki, T., Nakane, T., Kawamoto, A., Hojo, H., Kurisu, G. & Thakuria, R. (2023). *Cryst. Growth Des.* **23**, 5821–5826.

[bb12] Gomollón-Bel, F. (2019). *Chem. Int.* **41**, 12–17.

[bb13] Howard, J. L., Cao, Q. & Browne, D. L. (2018). *Chem. Sci.* **9**, 3080–3094. 10.1039/c7sc05371a PMC5933221 29780455

[bb14] Huang, Z., Ge, M., Carraro, F., Doonan, C., Falcaro, P. & Zou, X. (2021). *Faraday Discuss.* **225**, 118–132. 10.1039/d0fd00015a 33118574

[bb15] Ishi-i, T., Tanaka, H., Koga, H., Tanaka, Y. & Matsumoto, Y. (2020). *J. Mater. Chem. C*, **8**, 12437–12444.

[bb16] Kitagawa, H., Ohtsu, H. & Kawano, M. (2013). *Angew. Chem. Int. Ed.* **52**, 12395–12399. 10.1002/anie.201306776 24115515

[bb18] Marchetti, D., Guagnini, F., Lanza, A. E., Pedrini, A., Righi, L., Dalcanale, E., Gemmi, M. & Massera, C. (2021). *Cryst. Growth Des.* **21**, 6660–6664.

[bb17] Marchetti, D., Portone, F., Mezzadri, F., Dalcanale, E., Gemmi, M., Pedrini, A. & Massera, C. (2022). *Chem. A Eur. J.* **28**, e202202977. 10.1002/chem.202202977 PMC10092063 36161363

[bb19] Mugnaioli, E., Gorelik, T. & Kolb, U. (2009). *Ultramicroscopy*, **109**, 758–765. 10.1016/j.ultramic.2009.01.011 19269095

[bb53] Nannenga, B. L. & Gonen, T. (2014). *Curr. Opin. Struct. Biol.* **27**, 24–31. 10.1016/j.sbi.2014.03.004 PMC5656570 24709395

[bb20] Natarajan, R., Bridgland, L., Sirikulkajorn, A., Lee, J.-H., Haddow, M. F., Magro, G., Ali, B., Narayanan, S., Strickland, P., Charmant, J. P. H., Orpen, A. G., McKeown, N. B., Bezzu, C. G. & Davis, A. P. (2013). *J. Am. Chem. Soc.* **135**, 16912–16925. 10.1021/ja405701u PMC3880060 24147834

[bb54] Nederlof, I., van Genderen, E., Li, Y.-W. & Abrahams, J. P. (2013). *Acta Cryst.* D**69**, 1223–1230. 10.1107/S0907444913009700 PMC3689525 23793148

[bb51] Niebel, H., van den Berg, C., Steinfeld, G., van Veen, A. & Tuohimaa, T. (2021). European Patent Office. EP21191210.0.

[bb21] Palatinus, L., Brázda, P., Jelínek, M., Hrdá, J., Steciuk, G. & Klementová, M. (2019). *Acta Cryst.* B**75**, 512–522. 10.1107/S2052520619007534 32830709

[bb22] Palatinus, L., Corrêa, C. A., Steciuk, G., Jacob, D., Roussel, P., Boullay, P., Klementová, M., Gemmi, M., Kopeček, J., Domeneghetti, M. C., Cámara, F. & Petříček, V. (2015). *Acta Cryst.* B**71**, 740–751. 10.1107/S2052520615017023 26634732

[bb23] Petříček, V., Dušek, M. & Palatinus, L. (2014). *Z. Kristallogr. Cryst. Mater.* **229**, 345–352.

[bb24] Sasaki, T., Nakane, T., Kawamoto, A., Nishizawa, T. & Kurisu, G. (2023). *CrystEngComm*, **25**, 352–356.

[bb52] Sheldrick, G. M. (2008). *Acta Cryst.* A**64**, 112–122. 10.1107/S0108767307043930 18156677

[bb25] Sheldrick, G. M. (2015). *Acta Cryst.* A**71**, 3–8.

[bb26] Sieger, P., Werthmann, U. & Saouane, S. (2023). *Eur. J. Pharm. Sci.* **186**, 106447. 10.1016/j.ejps.2023.106447 37044200

[bb27] Simoncic, P., Romeijn, E., Hovestreydt, E., Steinfeld, G., Santiso-Quiñones, G. & Merkelbach, J. (2023). *Acta Cryst.* E**79**, 410–422. 10.1107/S2056989023003109 PMC10162091 37151820

[bb28] Wang, Z., Sikdar, N., Wang, S.-Q., Li, X., Yu, M., Bu, X.-H., Chang, Z., Zou, X., Chen, Y., Cheng, P., Yu, K., Zaworotko, M. J. & Zhang, Z. (2019). *J. Am. Chem. Soc.* **141**, 9408–9414. 10.1021/jacs.9b04319 31117669

[bb29] Woods, J. F., Gallego, L., Maisch, A., Renggli, D., Cuocci, C., Blacque, O., Steinfeld, G., Kaech, A., Spingler, B., Vargas Jentzsch, A. & Rickhaus, M. (2023). *Nat. Commun.* **14**, 4725. 10.1038/s41467-023-40475-8 PMC10406840 37550281

[bb30] Xu, H., Lebrette, H., Clabbers, M. T. B., Zhao, J., Griese, J. J., Zou, X. & Högbom, M. (2019). *Sci. Adv.* **5**, eaax4621. 10.1126/sciadv.aax4621 PMC6685719 31457106

