# Supplementary material for: 3D electron diffraction analysis of a novel, mechanochemically synthesized supramolecular organic framework based on tetra­kis-4-(4-pyridyl)phenyl­methane
Source: Acta Crystallogr B Struct Sci Cryst Eng Mater. 2023 Sep 26;79(Pt 6):432–6. doi: 10.1107/S2052520623007680 (PMC10833357; doi:10.1107/S2052520623007680)
Supplement: Supplementary file 3 [file b-79-00432-sup3.pdf]

Acta Cryst B
STRUCTURAL SCIENCE
CRYSTAL ENGINEERING
MATERIALS

Volume 79 (2023)

Supporting information for article:

3D Electron Diffraction analysis of a novel, mechanochemically-synthesized SOF based on tetrakis-4-(4-pyridyl) phenylmethane

Danilo Marchetti, Alessandro Pedrini, Chiara Massera, Moussa Faye Diame, Christian Jandl, Gunther Steinfeld and Mauro Gemmi

S1. Synthetic Procedures

The **TPPM** synthesis was carried out following the procedure reported in the literature (Kitagawa *et al.*, 2013).

The mechanochemical reactions were conducted with the addition of a small amount of liquid; this synthetic approach is called "Liquid Assisted Grinding" (LAG). The amount of liquid added to the reaction mixture and the total mass of the solid fraction is related to the parameter η (Friščić *et al.*, 2009).

S1.1. Mechanochemical Synthesis of TPPM·BnOH

TPPM (29.5 mg, 0.047 mmol) and BnOH (18 µL, 0.17 mmol) were placed into an agate mortar of 6 cm in diameter. The mixture was manually ground with a pestle for 20 min (η = 0.61) and then the mechanochemical crude was left in an open container for 1 hour to evaporate the remaining traces of LAG additive.

S2. Powder X-ray Diffraction (PXRD)

Manually selected points were used to describe the background, single crystal data (3D ED) were used to define the unit cell, and cyclic refinements on the entire dataset were used to generate the profile parameters. The peak profile was modelled as a Pseudo-Voight function, corrected due to axial divergence asymmetry and it is cut outside 20*FMWH range. The refinement converged to Rp = 5.77%, wRp = 7.68% and GOF = 2.87 (Figure S1).

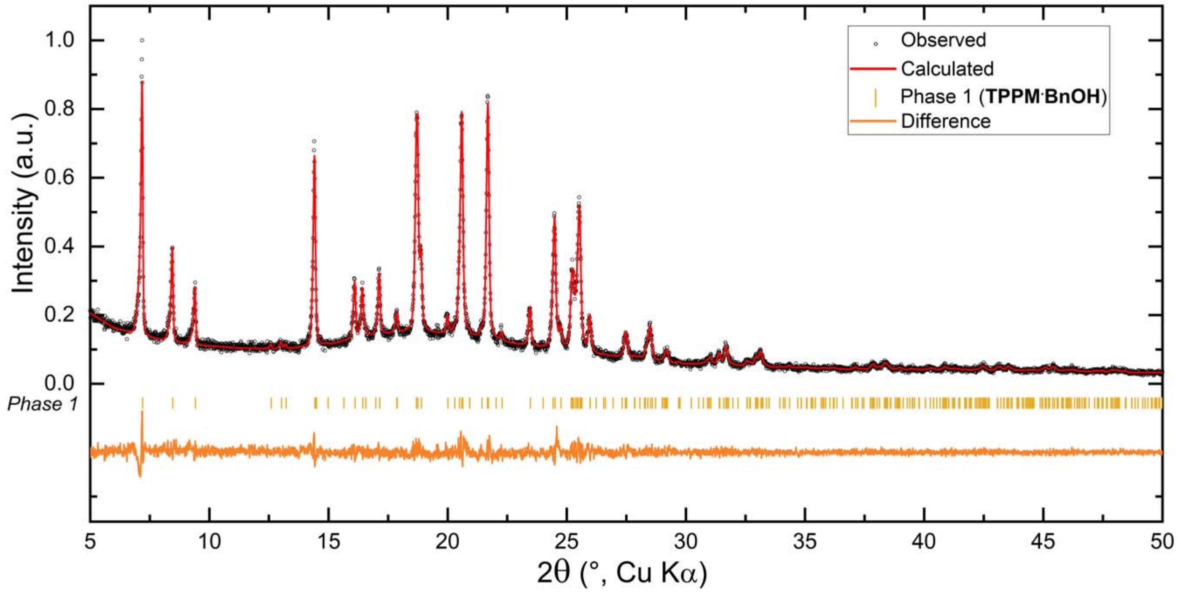

Figure S1 Profile fit from LeBail refinement on **TPPM·BnOH**. The shown range is limited to 2θ values of 5-50 for clarity, whereas the refinement was carried out in the range 5-75°. The refinement converged to Rp = 5.77%, wRp = 7.68% and GOF = 2.87.

Table S1 Unit cell parameters obtained from the LeBail Refinement on the powder X-ray diffraction data (PXRD).

	PXRD
a (Å)	28.469(2)
b (Å)	7.0652(4)
c (Å)	21.774(1)
α (°)	90
β (°)	120.481(4)
γ (°)	90
Vol (Å^3)	3774.33(3)

The indexed cells present a mC Bravais class.

S3. 3D Electron diffraction

S3.1. Sample preparation

The preparation of a sample suitable for 3D ED analysis was conducted using directly the mechanochemical reaction crude (see S1.2). The nanocrystalline powder of **TPPM·BnOH** was placed on a microscope glass and a TEM sample grid (Cu square grid, 300 mesh, holey carbon film) was gently pressed on it.

S3.2. Electron Diffractometer

3D electron diffraction analysis and scanning transmission electron microscopy imaging have been carried out on an ELDICO ED-1 electron diffractometer. A photograph of the setup is shown below. The electron gun is made with a self-ramping up beam system having a thermionic LaB_6 source which produces a 160 keV electron beam. The beam is shaped by multiple condenser lenses; however, no lens is located close to the sample chamber and no projector lenses are used to project the diffraction pattern onto the detector (Niebel *et al*. 2021). The condenser lenses are accompanied by several deflectors and among those is a pair of scanning coils that allow STEM imaging. There is only one aperture for both applicable modes, STEM imaging and diffraction. In STEM mode a focussed beam with diameters from 30-40 nm can be realized. The distance of the last condenser lens to the sample is ca. 35 mm, which was designed to leave space for further attachments, but also to have a rather large focal length, which means that even out of the focal plane a remarkable depth and sharpness of the images can be achieved, e.g. when images with a large field of view of a tilted or bent grid are recorded. In diffraction mode (parallel beam) recommended beam diameters are in the range from 300 to 950 nm with beam divergences in the range from 0.15 to 0.05 mrad, respectively.

The goniometer, which can mount a standard TEM grid, is equipped with four linear axes and one rotation axis which is vertical (Garbuglia *et al*. 2022). The construction of the goniometer allows to bring any position on a mounted grid into the point of intersection between rotation axis and the diffraction mode's electron beam (the usual eucentric point in a standard TEM). The movement of the goniometer (especially the sample rotation) is not restricted by any close-by optical elements. The detector is a hybrid pixel detector (Dectris QUADRO) which allows for continuous rotation experiments by its fast readout and neglectable dead time of 100 ns. Due to the fixed sample to detector distance and to the absence of any projection system compared to a TEM, once the detector distance is calibrated with a standard, no daily calibration is necessary and quite precise undistorted measurements of the reciprocal space geometry can be performed retrieving precise unit cell parameters.

The crystals suitable for the 3D ED analysis are searched through STEM imaging and their crystal quality is preliminarily checked with a single diffraction pattern, placing the beam on the crystal of

interest. During the analysis, the diffraction patterns are collected while the crystal is rotating, in accordance with the continuous-rotation data collection protocol.

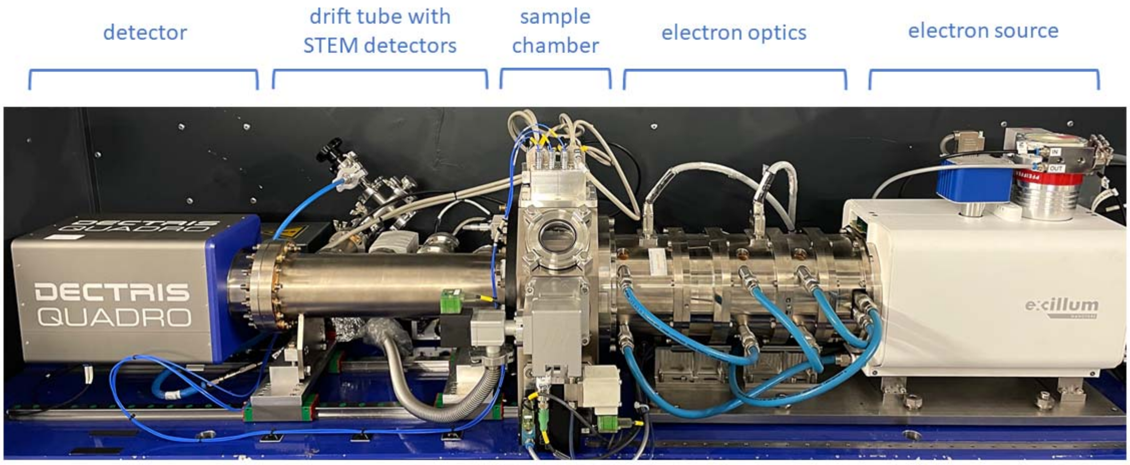

Figure S2 Photograph of the ELDICO ED-1 electron diffractometer setup. The sample chamber has an octagonal shape with several ports, of which the goniometer connection, a window, and the load lock for vacuum transfer are visible.

S3.3. Data Collection and Reduction

Electron diffraction data were collected on different microcrystals illuminated with a parallel electron beam of 750 nm in diameter. The diffraction patterns were recorded with a continuous rotation data collection protocol (cRED), which consists of a continuous scan of the reciprocal space, in this case, up to a maximum of 109° with an angular integration of 0.50° and one second of exposure time per frame. The position of the object on the TEM grid was assessed through STEM imaging. The eucentric height and the in-plane stability of the crystal were corrected through specific routines implemented in the software integrated with the instrument. Three different crystals were analyzed in total, and two data collections were performed in different regions of the Crystal 2.

The collected diffraction data of each crystal were separately handled using the PETS2 software (Palatinus *et al.*, 2019). The reflection data were indexed also determining the orientation matrix, subsequently the optimization of the frame orientation was performed and the reflection intensities were integrated. Furthermore, the diffraction data were combined using the PETS2 merging tool, which increased the completeness of the reflection data. The reflection intensities were treated with Jana2020 and the symmetrically equivalent reflections were averaged. The merged reflection dataset was used for the *ab-initio* structure determination, performed by Standard Direct Methods using the SHELXT software (Sheldrick, 2015). The data were initially refined with a fully kinematical approximation, which consists in neglecting the dynamical scattering phenomena and assuming that I_{hkl} is directly proportional to $|F_{hkl}|^2$. The least-squares refinement was performed with the software SHELXL-2014 (Sheldrick, 2008) interfaced with Olex2 (Dolomanov *et al.*, 2009). The position of the solvent molecule

was assessed from the calculation of the difference electrostatic potential map, with a resolution of 0.1 Å. The solvent molecules resulted disordered, so they were refined as a rigid body with an occupancy of 0.25. The amount of BnOH molecules inferred from the refined occupancy is in agreement with the calculated number of electrons from the solvent mask calculation. The solvent mask was calculated with Olex2 on the crystal structure with unmodelled BnOH molecules (Figure S3). The calculation led to 12 electrons per asymmetric unit (theoretical number: 14.5 electrons).

For the dynamical refinement, the reflections were integrated considering a rotation semiangle ($\Delta\alpha$) of 0.25°, since it should be one-half of the angular integration step. The integrated intensities were combined in virtual frames with the PETS2 software. Each virtual frame is composed of the sum of 8 experimental frames (N_F), for a covered semi-tilt range of 2° ($\Delta\alpha_v = N_F\Delta\alpha$), and the number of overlapping frames (N_O) was imposed to 4. The dynamical refinement was conducted on the crystal 2 and crystal 3 data, considering the unit cell obtained from the crystal 2^b data. The dataset from crystal 1 was omitted due to quality reasons. The refinement was conducted simultaneously on the crystal 2 and 3 reflection files using the Jana2020 software. The dynamical refinement was performed after an initial optimization of the crystal thickness and frame geometry. The crystal structure was then refined together with the crystal thickness of the respective crystals of each data collection.

Table S2

Angular coverage of the data collections performed on the analysed microcrystals. The reported values were obtained after a frame-by-frame fine tuning of the geometrical parameters, performed with the PETS2 software.

	Crystal 1	Crystal 2^a	Crystal 2^b	Crystal 3
Starting Angle (°)	-69.90	-30.19	-69.73	-3.05
Ending Angle (°)	28.97	39.14	39.39	64.79
Angular Range (°)	98.87	69.33	109.11	67.85
Estimated total dose (e/Å^2)	2.75	1.92	3.03	1.89

*Two different data collections have been conducted on crystal 2. 2^a: first data collection on crystal 2; 2^b: second data collection on crystal 2. Estimated total dose based on 0.0139 e A^{-2} s^{-1} dose rate at an emission current of 20 μA.

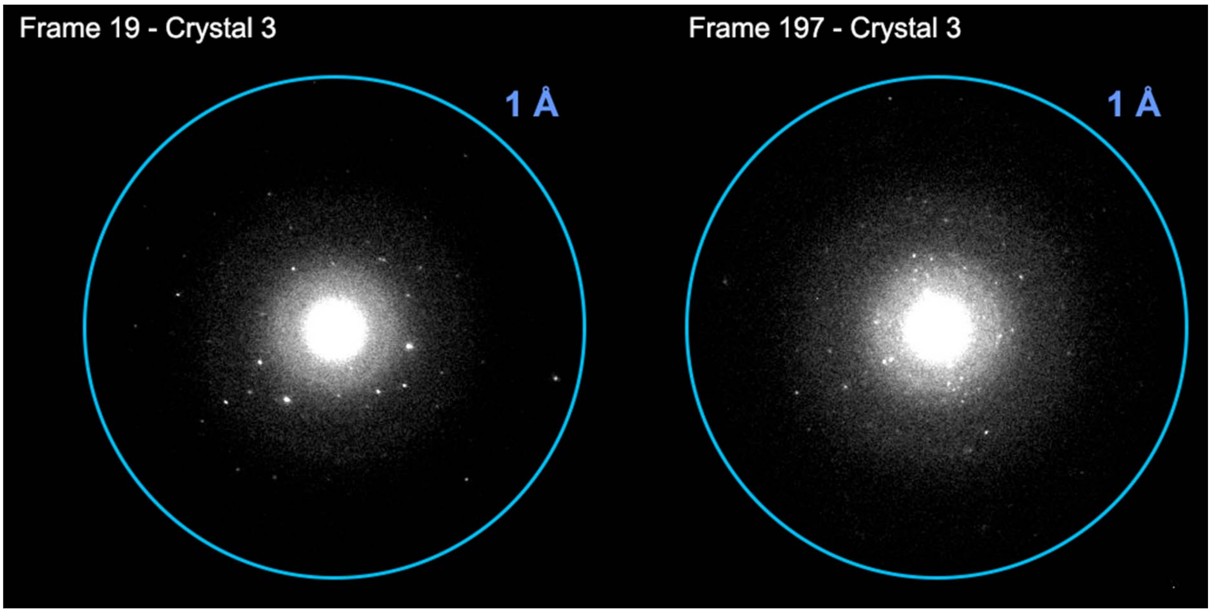

Figure S3 Electron diffraction patterns from the data collection on crystal 3. The figure shows two distinct diffraction patterns at the start (left) and end (right) of the data collection. The blue circle highlights the resolution of 1Å.

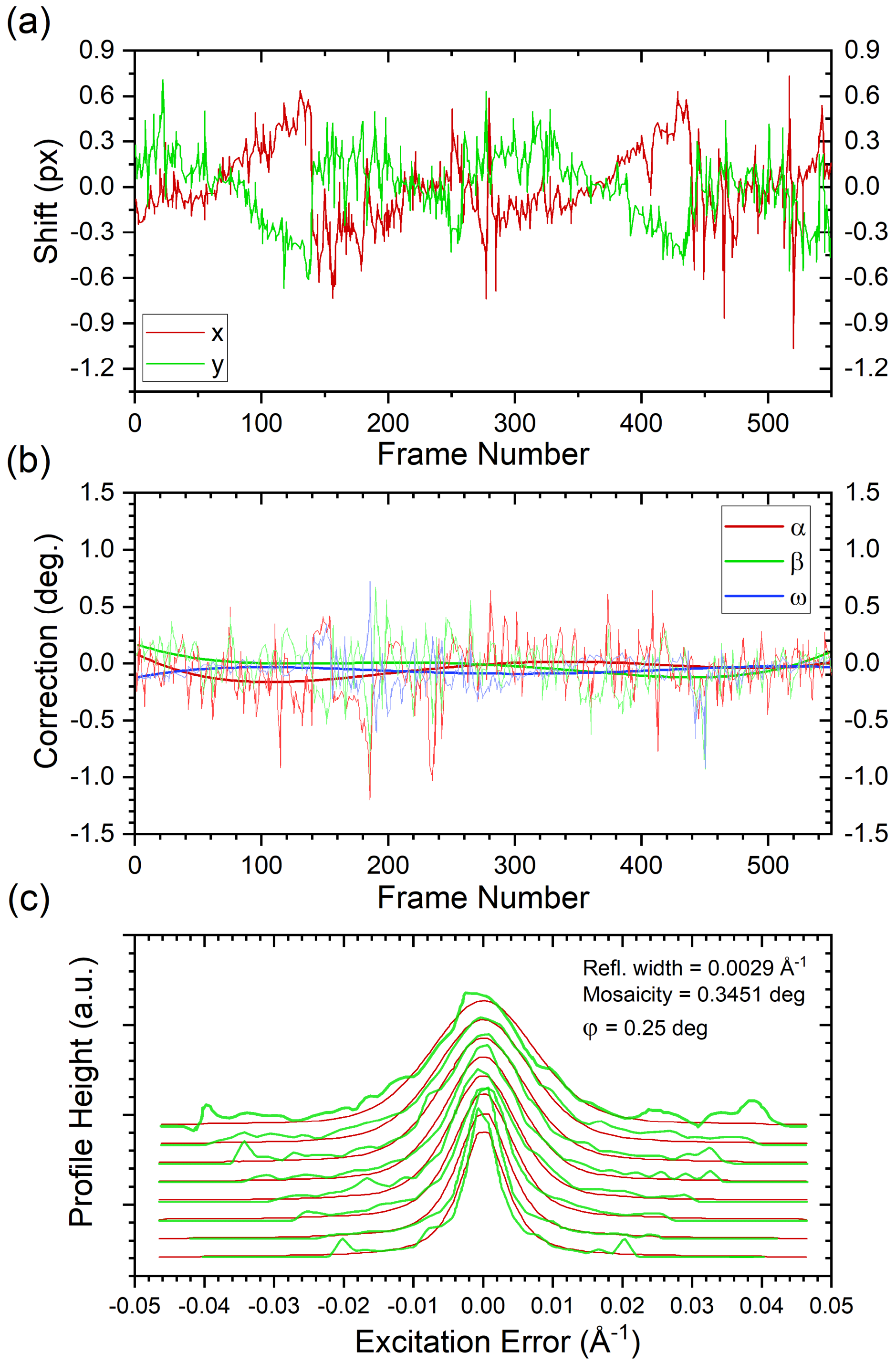

Figure S4 (a): Plot of the shift of the pattern centre. From the centre shift it is possible to distinguish the four datasets used in the data reduction process. (b): Plots of the geometrical corrections applied to each diffraction pattern. ω: angle comprised between the tilt axis and the positive horizontal axis of the image. α: tilt angle. β: second tilt angle. (c): Plot of rocking curve (RC) profiles of the merged datasets. The lowest curve corresponds to the average RC in the range 0.2 to 0.3 Å^{-1}, the next curves are obtained after a step of 0.1 Å^{-1} until 0.9 to 1 Å^{-1}. The red curve is the calculated RC profile while the green line represents the experimental profile.

Table S3

Unit cell parameters obtained from the indexed data of the analysed crystals.

	Crystal 1	Crystal 2[a]	Crystal 2[b]	Crystal 3	Merged
a (Å)	27.98(1)	27.95(1)	27.946(7)	27.90(1)	27.907(7)
b (Å)	7.014(1)	7.019(1)	7.0236(5)	7.023(2)	7.0195(7)
c (Å)	21.72(1)	21.555(7)	21.610(3)	21.57(1)	21.592(3)
α (°)	90	90	90	90	90
β (°)	118.81(3)	118.62(3)	119.031(14)	118.67(4)	118.821(12)
γ (°)	90	90	90	90	90
Vol (Å^3)	3734.0(4)	3712.5(3)	3708.7(12)	3705.1(4)	3705.9(12)
Indexed (%)	59.51	76.30	67.52	65.16	68.80
R_{int}(obs) (%)	12.01	11.54	9.93	11.22	24.06
Completeness (%)	73	51	78	69	82
Redundancy	2.44	1.99	2.51	2.74	2.61

The indexed cells belong to an mC Bravais class.

Table S4 Comparison between the unit cell parameters from 3D ED analysis and from the LeBail refinement on PXRD data.

	3D ED **TPPM-E**	3D ED **TPPM·0.5BnOH**	PXRD **TPPM·BnOH**
a (Å)	31.4066	27.907(7)	28.469(2)
b (Å)	7.1129	7.0195(7)	7.0652(4)
c (Å)	22.063	21.592(3)	21.774(1)
α (°)	90	90	90
β (°)	133.175	118.821(12)	120.481(4)
γ (°)	90	90	90
Vol (Å^3)	3594.3(4)	3705.9(12)	3774.33(3)

The indexed cells belong to an mC Bravais class.

Table S5

Crystal data and structure refinement for the **TPPM·0.5BnOH** phase from the merged datasets at the kinematical theory level.

Empirical formula	$C_{48.5}H_{35.5}N_4O_{0.5}$
Formula weight	682.35
Temperature/K	293(2)
Crystal system	Monoclinic
Space group	C2/c
a/Å	27.907(7)
b/Å	7.0195(5)
c/Å	21.592(3)
α/°	90
β/°	118.821(14)
γ/°	90
Volume/$Å^3$	3705.9(12)
Z	4
$\rho_{calc}g/cm^3$	1.223
F(000)	1434
Crystal size/mm^3	Crystal 1: 0.0015 × 0.00055 × 0.00032<br>Crystal 2: 0.0042 × 0.0015 × 0.00058<br>Crystal 3: 0.001 × 0.0004 × 0.00025
Radiation	Electrons (λ = 0.02851 Å)
2θ range for data collection/°	0.16 to 1.922
Index ranges	$-32 \le h \le 32, -8 \le k \le 8, -22 \le l \le 25$
Reflections collected	7491
Independent reflections	2636 [R_{int}= 0.2406, R_{sigma}= 0.1983]
Data/restraints/parameters	2636/54/132
Goodness-of-fit on F^2	1.958
Final R indexes [I>=2σ (I)]	R_1= 0.2747, wR_2= 0.5723
Final R indexes [all data]	R_1= 0.3073, wR_2= 0.5886

[a] $R_1 = \Sigma||F_o| - |F_c||/\Sigma|F_o|$, $wR_2 = [\Sigma[w(F_o^2-F_c^2)^2]/\Sigma[w(F_o^2)^2]]^{1/2}$

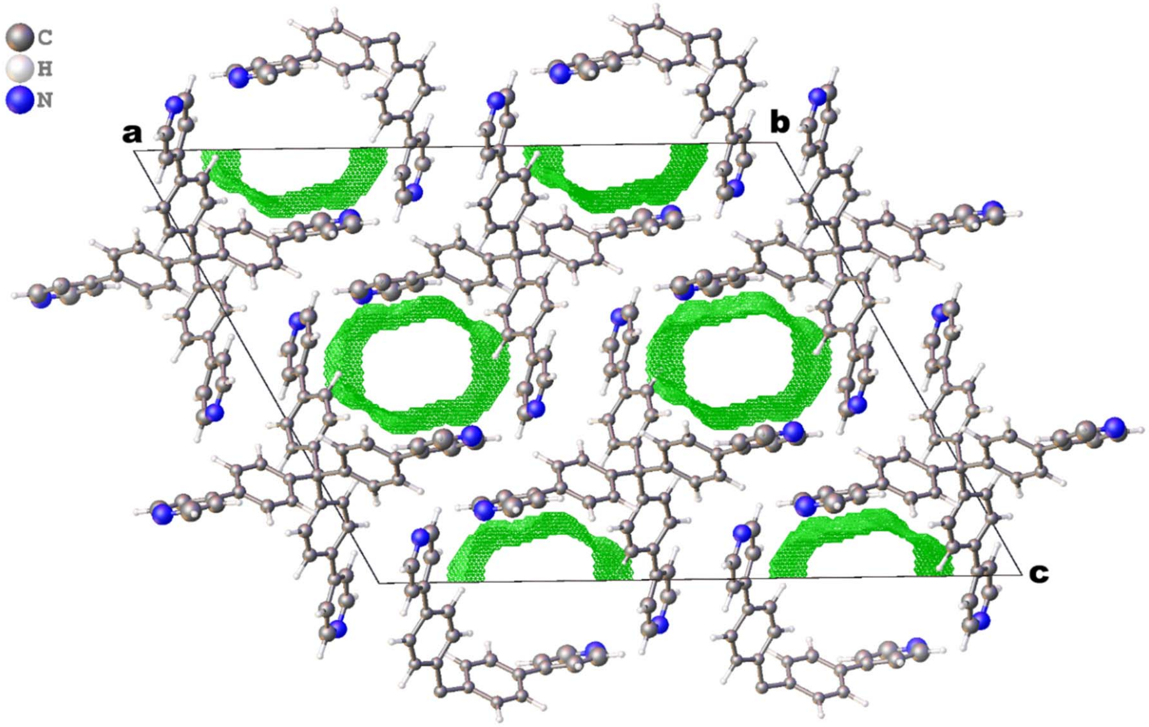

Figure S5 Representation of the solvent mask as a green surface along the crystallographic *b*-axis direction.

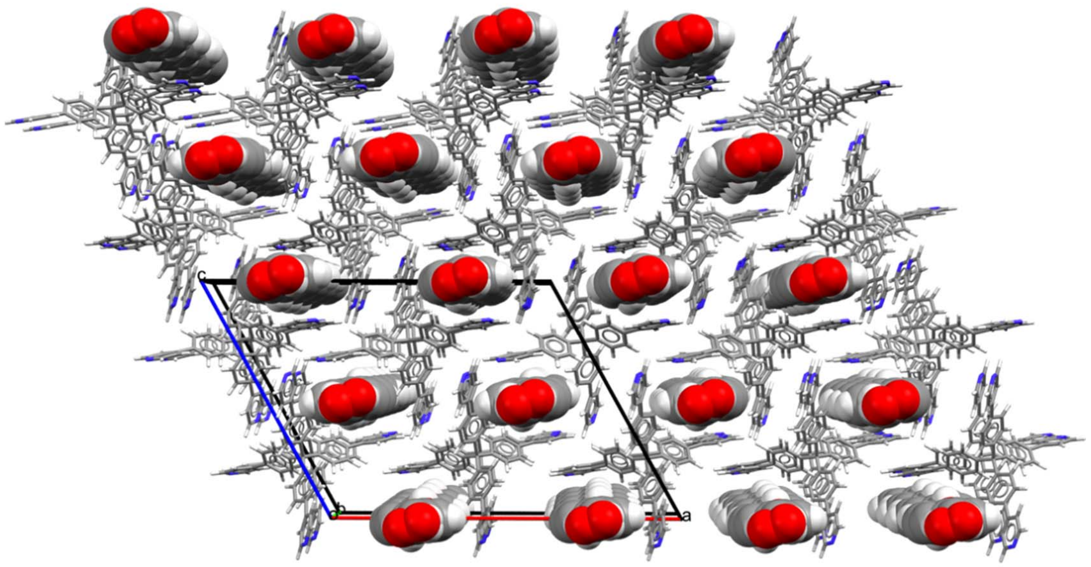

Figure S6 The **TPPM·0.5BnOH** structural model oriented along the crystallographic *b*-axis. The oxygen atoms are represented in red, carbon atoms in grey, nitrogen atoms in blue and hydrogen atoms in white. The disordered solvent molecules are shown in space filling mode.

Table S6

Crystal data and structure refinement for the **TPPM·0.5BnOH** phase from the dataset simultaneously refined at the dynamical theory level.

Empirical formula	$C_{48.5}H_{35.5}N_4O_{0.5}$
Formula weight	682.35
Temperature/K	293(2)
Crystal system	Monoclinic
Space group	C2/c
a/Å	27.946(7)
b/Å	7.0236(5)
c/Å	21.610(3)
α/°	90
β/°	119.031(14)
γ/°	90
Volume/Å^3	3708.7(12)
Z	4
ρ_{calc}g/cm^3	1.223
F(000)	1434
Crystal size/mm^3	Crystal 1: 0.0015 × 0.00055 × 0.00032<br>Crystal 2: 0.0042 × 0.0015 × 0.00058<br>Crystal 3: 0.001 × 0.0004 × 0.00025
Radiation	Electrons (λ = 0.02851 Å)
2θ range for data collection/°	0.16 to 1.922
Index ranges	$-26 \le h \le 27$, $-6 \le k \le 6$, $-21 \le l \le 20$
Reflections collected	15678
Independent reflections	3291 [R_{int}= 0.2406, R_{sigma}= 0.1983]
Data/restraints/parameters	3291/50/210
Goodness-of-fit on F^2	3.9081
Final R indexes [I>=3σ (I)]	R_1= 0.1442, wR_2= 0.1479
Final R indexes [all data]	R_1= 0.2031, wR_2= 0.1532

[a] $R_1 = \Sigma \| F_o | - | F_c \| / \Sigma | F_o |$, $wR_2 = [\Sigma[w(F_o^2-F_c^2)^2]/\Sigma[w(F_o^2)^2]]^{1/2}$

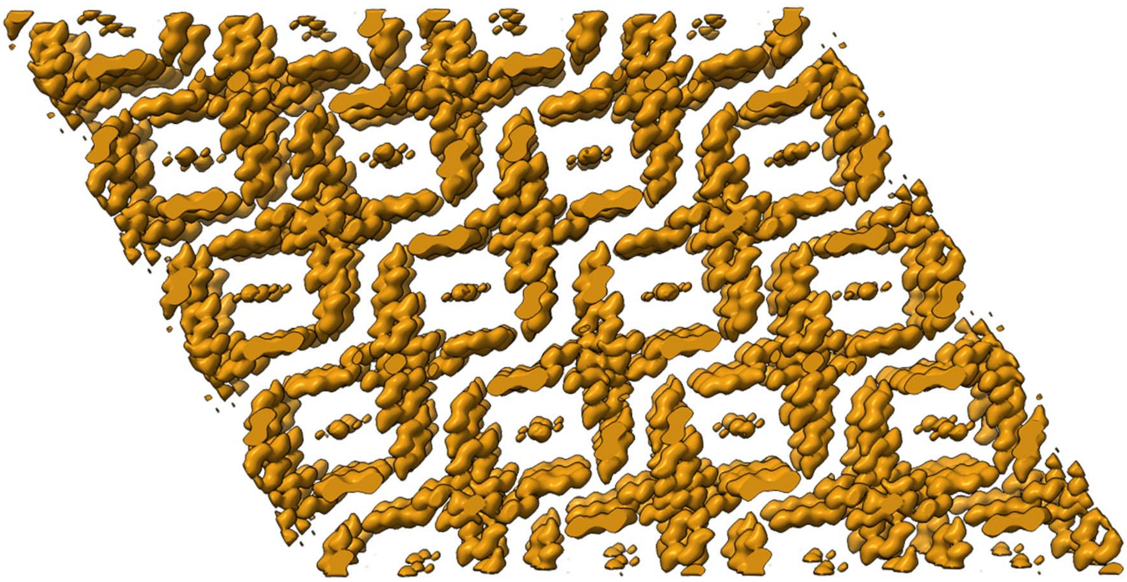

Figure S7 Electrostatic potential map of the **TPPM** structural model, visualized as isosurface within the unit cell borders. The potential map is calculated from the dynamically refined structure by Fourier map calculations.

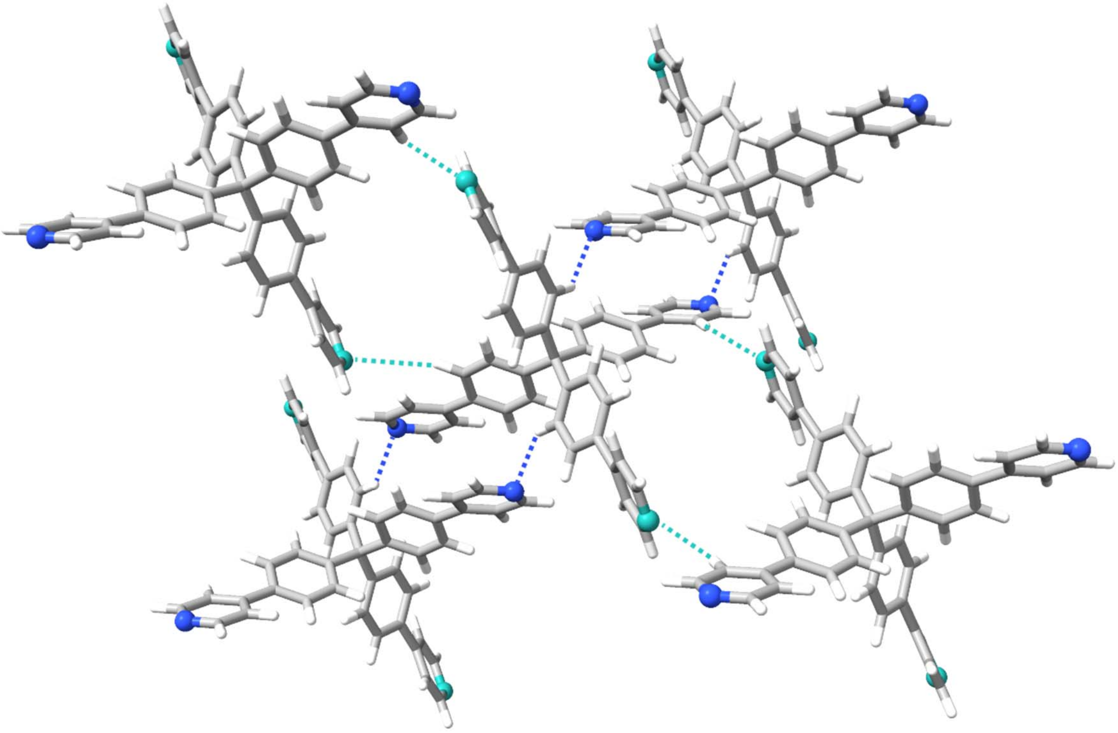

Figure S8 View of the main C-H···N interactions in **TPPM·0.5BnOH**. The N atoms involved in the interaction are represented as blue (N1A) and turquoise (N1B) spheres. The solvent molecules are

omitted for clarity. The interactions N1A···C3B and N1B···C9A are represented by blue and turquoise dashed lines, respectively. N1A···C3B: 3.72(3) Å; N1B···C9A: 3.66(3) Å; N1A···H3B-C3B: 140.79°; N1B···H9A-C9A: 130.93°.

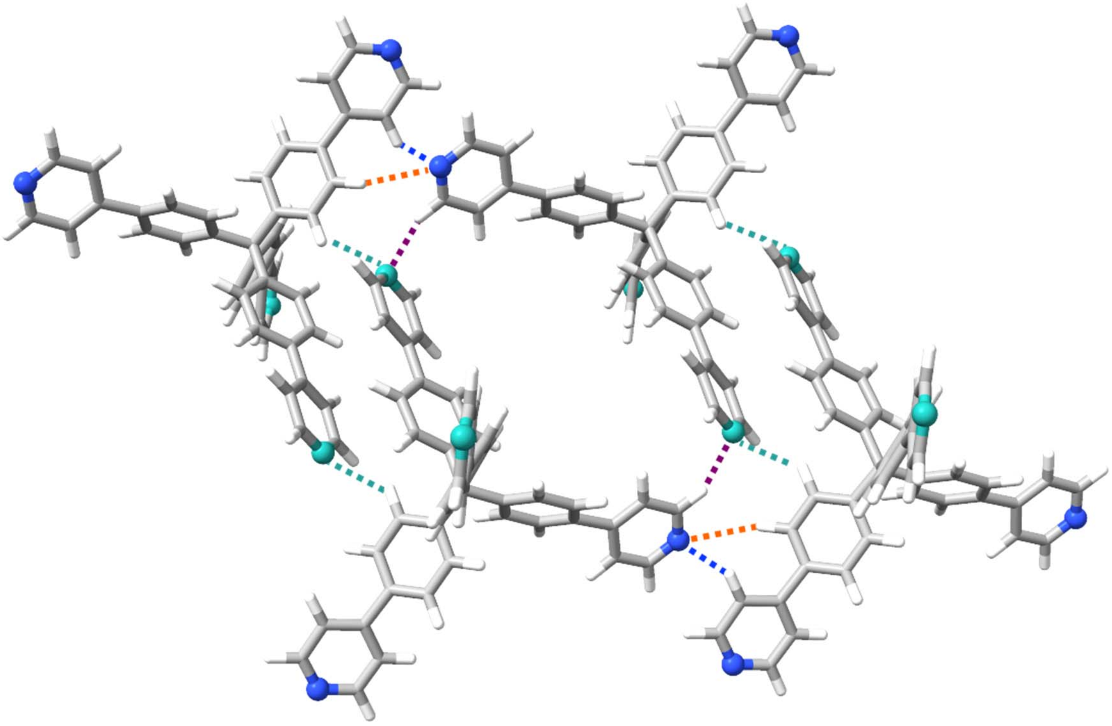

Figure S9 View of the main C-H···N interactions in **TPPM·0.5BnOH**. The N atoms involved in the interaction are represented as blue (N1A) and turquoise (N1B) spheres. Solvent molecules have been omitted for clarity. The interactions N1A···C3B, N1B···C9A, N1A···C10B and N1B···C4B are shown by blue, turquoise, purple and orange dashed lines, respectively. N1A···C10B: 3.55(3) Å; N1B···C4B: 4.02(4) Å; N1A···H10B-C10B: 150.17°; N1B···H4B-C4B:163.68°.

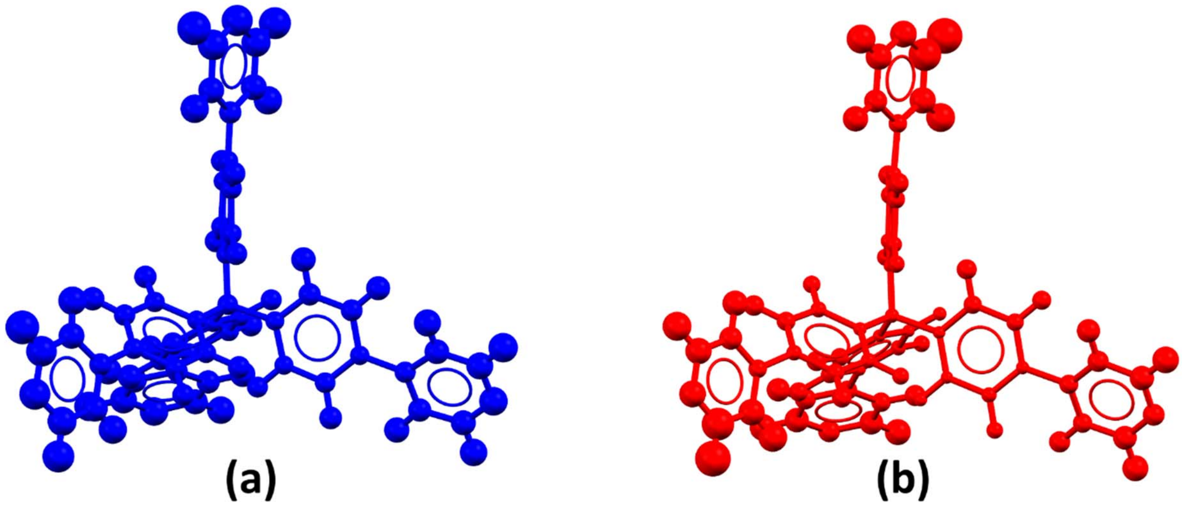

Figure S10 View of the **TPPM** molecule in a ball a stick representation, in which the radius of the sphere is directly proportional to U_{iso} values. (a) Kinematical refinement; (b) Dynamical refinement.

S4. Thermogravimetric Analysis (TGA)

The thermogravimetric analysis of **TPPM·BnOH** was conducted on a Perkin Elmer Instrument, model TGA 8000. The desorption process of the guest molecules was followed in the temperature range 30-350°C with a heating rate of 10°C min^{-1} under nitrogen flux.

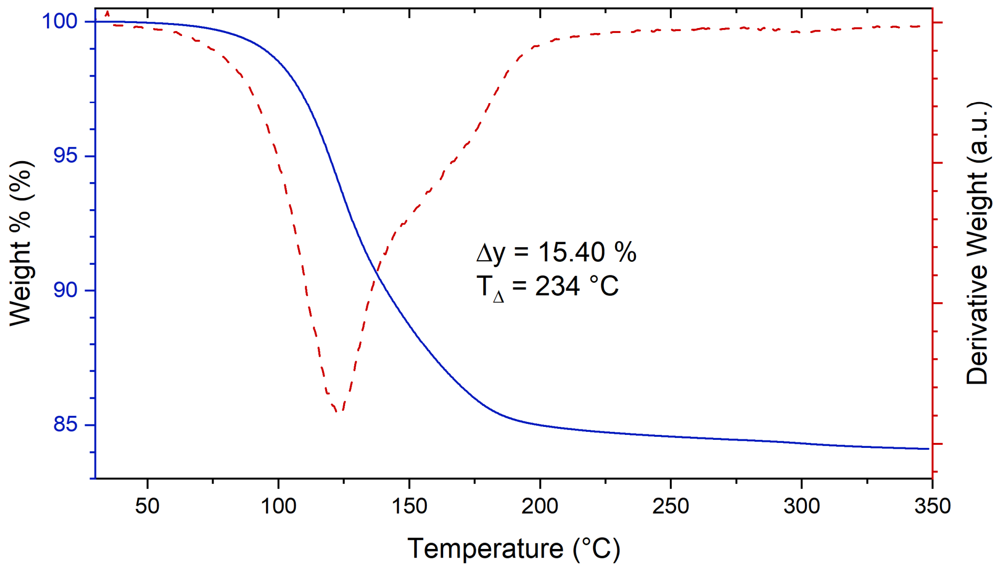

Figure S11 Thermogravimetric path recorded on the **TPPM·BnOH** crystal phase. The blue line corresponds to the weigh percentage on temperature, while the red dashed line is the first derivative of the weight percentage.

The solvate stoichiometry (SS^{TGA}) was calculated from the weight loss, to define the **TPPM**/BnOH ratio at atmospheric conditions (Figure S10). The SS^{TGA} of the **TPPM·BnOH** phase, at room conditions, resulted close to 1.

$$SS^{TGA} = \frac{\Delta W(\%) \cdot (MW_{TPPM} + MW_{Guest})}{100 \cdot MW_{Guest}}$$

Figure S12. Equation adopted for the calculation of the solvate stoichiometry(SS^{TGA}) from TGA data.

S5. NMR Characterization

The ^{1}H NMR spectra were collected in CD_2Cl_2 with few drops of methanol-d4, to obtain a clear solution of **TPPM**, on a Bruker AC400 Avance.

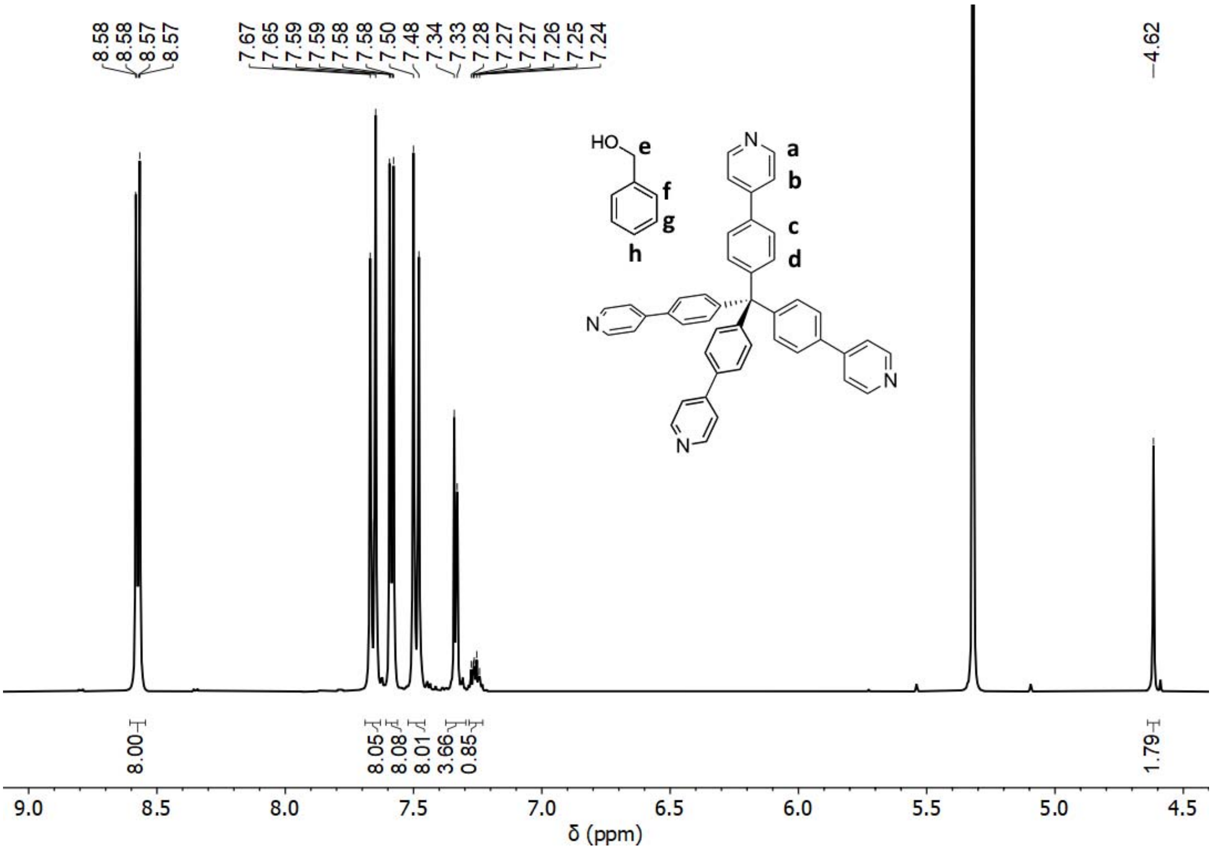

Figure S13 ^{1}H NMR spectrum of **TPPM·BnOH** (400 MHz, CD_2Cl_2) δ (ppm): 8.57 (dd, J_1 = 4.7 Hz, J_2 = 1.7 Hz, 8H, a), 7.66 (d, J = 8.6 Hz, 8H, c), 7.59 (dd, J_1 =4.8 Hz, J_2 = 1.7 Hz, 8H, b), 7.49 (d, J = 8.6 Hz, 8H, d), 7.33 (m, 4H, f + g), 7.26 (m, 1H, h), 4.62 (s, 2H, e).

$$SS^{NMR} = \frac{I_{BnOH} \cdot n_{TPPM}}{I_{TPPM} \cdot n_{BnOH}}$$

Figure S14. Equation adopted for the calculation of the solvate stoichiometry (SS^{NMR}) from ^{1}H NMR data. I_{BnOH} : integral associated to a diagnostic peak of the **BnOH** molecule; I_{TPPM} : integral associated to a **TPPM** diagnostic peak; n_{BnOH} : number of hydrogen atoms related to the **BnOH** diagnostic peak; n_{TPPM} : number of hydrogen atoms related to the **TPPM** diagnostic peak.

References

Friščić, T., Childs, S. L., Rizvi, S. A. A. & Jones, W. (2009). *CrystEngComm.* **11**, 418–426.

Garbuglia, F. & Steinfeld, G. (2022). *European Patent Office.* EP22192609.0.

Gemmi, M. & Lanza, A. E. (2019*). Acta Cryst.* **B75**, 495-504.

Kitagawa, H., Ohtsu, H. & Kawano, M. (2013). *Angew. Chem. Int. Ed.* **52**, 12395–12399.

Niebel, H., van den Berg, C., Steinfeld, G., van Veen, A. & Tuohimaa, T. (2021). *European Patent Office*. EP21191210.0.

Sheldrick, G. M. (2008). *Acta Crystallogr.* **A64** (1), 112–122.

Sheldrick, G. M. *SHELXT* – Integrated space-group and crystal-structure determination. (2015). *Acta Cryst.* **A71** (1), 3–8.

Palatinus, L., Brázda, P., Jelínek, M., Hrdá, J., Steciuk, G. & Klementová , M. (2019). *Acta Cryst.* **B75**, 512–522.
